# *De novo* whole transcriptome analysis of *Aeromonas hydrophila* isolated from the gut of an infected *Labeo rohita*

**DOI:** 10.3389/fmicb.2023.1247652

**Published:** 2023-09-14

**Authors:** Basanta Kumar Das, Vikash Kumar, Priyanka Das, Kausalya K. Nayak

**Affiliations:** ^1^Aquatic Environmental Biotechnology Division, ICAR-Central Inland Fisheries Research Institute, Barrackpore, West Bengal, India; ^2^Department of Zoology, K.B.D.A.V. College, Nirakarpur, Odisha, India

**Keywords:** *Aeromonas hydrophila*, transcriptome analysis, virulence genes, *Labeo rohita*, microbial management

## Abstract

*Aeromonas hydrophila* is a major generalist bacterial pathogen causing severe infections and mortalities in aquatic animals. Its genome, which was the first to be sequenced from the *Aeromonas* genus, may serve as a model for studying pathogenic mechanisms. To explore the pathogen-host fitness mechanism of bacterium, a comprehensive comparative transcriptome ecotype analysis of *A. hydrophila* isolated from the gut of *Labeo rohita* during infection was performed. Special characteristics in gene expression, gene ontology terms and expression of pathogenesis-associated genes, including genes encoding secreted proteins, candidate effectors, hydrolases, and proteins involved in secondary metabolite production were revealed. Among the database, 6,533 were gene ontology (GO) annotated, while 1,480 were not allocated in any GO terms. Investigation on GO illustrated that the articulated genes were improved with molecular function, cellular components, and biological processes. Further bioinformatics analysis identified the outer membrane protein genes (ompA, ompts, ompw, omp38, and omp48), cytotoxin, amylase, and lipase genes. Overall, this work allowed to designate, for the first time, a global view on the pathogenicity of *Aeromonas hydrophila* during infection. Furthermore, the study provides information on the fitness of *A. hydrophila*, a severe pathogen with a wide host range.

## Highlights

- The *de novo A. hydrophila* Whole transcriptome sequencing has been carried out using SOLiD sequencing platform.
- Transcriptomics analysis facilitated categorizing of biologically important genes as well as their functional correlation with fish infections caused by *A. hydrophila.*
- The significant genes, expressed in the specific state have been recognized subsequent to transcript annotation and gene ontology study.

## 1. Introduction

In recent decades, advances in understanding how pathogenic bacteria respond to host animal and biotic or abiotic environmental factors have been proposed in many important aquatic bacteria (Kumar et al., 2023a,b). Previous research mostly focused on characteristics host transcriptome analysis revealed the characteristics of conservation and specificity in the gene expression of host animals interacting with bacterial pathogens. For instance, the transcriptome of sturgeon fish, red swamp crayfish and Pacu infected with *Aeromonas hydrophila* provide a detailed insight on host genes expressed during bacterial infection (Das and Mukherjee, 1997; Jiang et al., 2018; Mastrochirico-Filho et al., 2020; Liu et al., 2021). In our recent work, the immune gene expression profile of *Labeo rohita* revealed the influence of *A. veronii* and *A. hydrophila* on host health, showing that some proteins and genes upregulated might be playing an important role in *host resistance during infection* (Kumar et al., 2022; Behera et al., 2023).

The water-borne *Aeromonas hydrophila* is a destructive and widespread aquatic pathogen widely distributed in both polluted and non-polluted water bodies causing significant economic losses worldwide and is one of the top 10 fish pathogenic bacteria based on scientific/economic importance in the aquaculture system (Kumar et al., 2022). Bacterial strain from genera *Aeromonas is a* gram-negative, facultative anaerobic, motile bacterium that is the causative agent of Motile Aeromonas septicemia (MAS). It is well known that *A. hydrophila* express diverse virulence factors including antigen-O, the presence of capsules, S-layer, exotoxins such as hemolysins and enterotoxins, exoenzymes such as lipase, amylase and protease and the type III secretion system involved in attacking the host fish and competing with environmental bacteria. The exotoxins, enterotoxins, exoenzymes and the type III secretion system digest their hosts more extensively than any other microbes and promote fish cell death to provide nutrients for the multiplication and colonization of these bacterial pathogens in the course of infection (Abdella et al., 2023). Previous work has suggested that presence of virulence factors, particularly those related to extracellular products, play an important role in the translocation of *Aeromonas* spp. in the epithelium, thus being broadly associated with bacterial virulence. The aerolysin, lipases and hiydrolipases are considered important virulence factors in *Aeromonas* spp. because they alter the structure of the cytoplasmic membrane of the host and thus exacerbate bacterial pathogenicity (Krovacek et al., 1995; Lye et al., 2007; Oliveira et al., 2012). However, we know little about regulators controlling gene expression, encoding enzymes in the metabolic pathways and gene ontology, which are curial in the virulence of bacteria.

RNA-sequencing (RNA-Seq) has become a very popular method for elucidating the molecular mechanisms involved in specific biological and disease processes. Using RNA-Seq, much research has been conducted on pathogen-host interactions (PHIs) in major fish diseases (Das P. et al., 2019; Das et al., 2020). Based on dynamic expression changes, RNA-Seq approaches identify genes that function in pathogen infection and adaption processes, such as pathogenicity, metabolism, signalling regulation, and response to complex environmental factors (El-Hossary et al., 2023; Lee et al., 2023). Currently, the genomes of *A. hydrophila* have been sequenced and the genomes have been compared with intraspecific subgroups of bacterium, but we know little about genome-wide spatiotemporal expression during *A. hydrophila* infection. In the present work, we recovered the cells of *A. hydrophila* from gut samples of Labeo rohita during infection. The transcriptome sequence information of *A. hydrophila* has been analyzed, highlighting the critical immunological genes. This will possibly escalate our perception of the relationships between genes and the transcriptome of *A. hydrophila* during pathogenesis in the host.

## 2. Materials and methods

### 2.1. Outbreak description

From mid-April 2022 to the beginning of May 2022, we investigated a case of severe mortality in juvenile size *Labeo rohita* (Length = 120.4 ± 6.8 mm, Weight = 12.51 ± 1.9 g) in the aquaculture farms of Moina, Purba Medinipur district (22^o^18’33 N, 87^o^50’20 E), West Bengal, India. In the aquaculture farms, mixed fish species, including *L. rohita*, *Cirrhinus mrigala*, *L. catla,* and *Hypopthalmichthys molitrix* were cultured. As the central nodal agency for fish disease surveillance, the team of researchers from ICAR-Central Inland Fisheries Research Institute, Kolkata, India visited the culture facility. During our visit, we estimated approximately 60% mortality in *L. rohita* through passive data collection. The fish were showing signs of disease, including extreme lethargy, hemorrhage, ulcer, discoloration, and redness in fins and all over the body surface. Suspecting a microbial infection, moribund juveniles of *L. rohita* showing clinical signs were transported to the laboratory, necropsied, and subjected to further microbiological tests.

### 2.2. Bacteria isolation, total RNA extraction and transcriptome library preparation

Diseased rohu fish (*Labeo rohita*) showing clinical signs, including ulcerated skin, muscle lesions, and tail and fin rot, are collected and sacrificed for bacterial isolation. The fish were anaesthetized using clove oil (Merck, Germany) at a 50 μL/L dose, and the gut sample was collected aseptically from the fish and transferred to Nutrient Broth (NB) (Himedia, India) and incubated for 24 h at 37°C at 200 rpm. The growth of morphologically similar colonies on Nutrient Agar (NA) plates indicates the presence of a single bacterial strain. A single colony was streaked onto freshly prepared NA plates and incubated at 37°C for 24 h to obtain a pure strain. Subsequently, the colony was transferred to NB, and after 24 h of incubation at 37°C, the bacterial strain stock culture was prepared in 30% glycerol and stored at-20°C until further use.

### 2.3. Acclimatization of experimental fish and challenge assay

Organization for Economic Cooperation and Development (OECD) guidelines were followed for the handling and care of experimental animals. The animal utilization protocol was approved by Institutional Animal Ethics Committee, ICAR-Central Inland Fisheries Research Institute, Kolkata, India, (IAEC/2021/04) for the experimental setup.

Healthy *L. rohita* (Length = 114.2 ± 2.5 mm, Weight = 12.1 ± 5.2 g) were purchased from a local fish hatchery. The fish appeared normal and healthy with no external clinical symptoms like ulcer, hemorrhage, descaling, discoloration, and redness in the body surface was used in the experiment. Additionally, the fish were randomly selected and screened for the possible presence of infectious microbes following the standard protocol (Johansen et al., 2006). The fish was acclimatized for 2 weeks in 200 L Fibre-reinforced plastic (FRP) tanks, supplied with proper commercial floating feed (CP: 30%, CL: 5%) @ 3–5% of body weight fed twice a day. The bacterial strain was cultured in 20 mL sterile Tryptone soya broth (TSB) in a 50 mL Erlenmeyer flask (Himedia, India) for 24 h at 28°C. The bacterial cells pellet was collected by centrifuging for 5 min at 5000 rpm and washed thrice with a sterile normal saline solution. Afterward, the pellets were resuspended in saline solution and the number of cells (CFU/ml) was estimated through the spread plate method. The experimental fish were intraperitoneally injected (20 numbers/ concentration) with 200 μL (1.2 × 10^1^, 1.2 × 10^2^, 1.2 × 10^3^, 1.2 × 10^4^, 1.2 × 10^5^, 1.2 × 10^6^ and 1.2 × 10^7^ CFU/mL) of the bacterial suspension. Control fish were injected with 200 μL of saline solution. Afterward, the fish were kept in an FRP tank and observed every 24 h until 120 h. To confirm Koch’s postulate, the bacteria were reisolated and identified from the liver, kidney, and blood of the moribund fish.

### 2.4. Bacteria identification by 16S rRNA gene and phylogenetic analysis

At first, the isolated strain was primarily distinguished as either Gram-negative or Gram-positive by the Gram-staining method. Later, the genomic bacterial DNA was isolated following the standard Sarkosyl method (Kumar et al., 2022). In brief, the quality of the DNA extracted was checked on agarose gel (1%) and quantified using Nano-drop (Eppendorf, Germany). The 16S rRNA gene was amplified by Gene Amp PCR system 9,700 thermal cycler (Applied Biosystems, Foster City, CA) using universal bacterial primers. 50 μL of the PCR reaction mixture consisted of 10× PCR buffer (5 μL), 50 mM MgCl_2_ (1 μL), 10 mMdNTP (1 μL), 10 pmol of each primer (1 μL), isolated genomic DNA (100 ng), and Taq DNA polymerase (1 U). The PCR condition comprised of 2 min at 95°C initial denaturation, 35 cycles of 94°C for 30 s denaturation, 52°C for 60 s annealing and 72°C for 90 s extension and 7 min at 72°C of final extension. Subsequently, the PCR-amplified products were visualized on agarose gel (1.8%) (Behera et al., 2017). The amplified gene was sequenced in forward and reverse directions using ABI 373xl capillary sequencer (Applied Biosystem, Foster City, CA). Contig was prepared by aligning forward and reverse sequences using DNA baser 7.0.0. The amplified 16S rRNA gene from the strain was sequenced and submitted to GenBank, and accession number JF330411 was obtained. The BLAST analysis against the non-redundant database showed >99% identity with *Aeromonas hydrophila* (GenBank Accession Numbers: MT384379 and CP046954).

### 2.5. Bacterial RNA extraction for transcriptome analysis

For RNA extraction, bacterial cells were harvested through centrifugation at 5000 rpm for 2 min, then washed with DEPC-treated water. The collected cells were frozen in liquid nitrogen, and we proceeded to isolate total RNA. Total RNA was isolated by a customised protocol combining the RaFlex total RNA isolation kit (Genei, India) and the Xcelgen RNA isolation kit (Xcelris Labs, India). The total RNA (1 μg) was estimated on a 1% denaturing agarose gel. The total RNA (4 ng/μl) was analysed on an Agilent Pico chip on an Agilent Bioanalyzer (Agilent, United States). The bioanalyzer profile of total RNA with a RIN value of 9 was obtained, which assured the presence of intact RNA in the sample. Total RNA of *A. hydrophila* isolated from bacterial cells was applied to enhance the RNA transcripts by collectively depleting ribosomal RNA molecules (rRNA) by applying MICROB Express bacterial mRNA purification kit (ThermoFisher Scientific, United States). Following rRNA-depleted mRNA isolation, the samples were arranged to form a transcriptome library employing the SOLiD Total RNA Seq kit (ThermoFisher Scientific, United States). The rRNA depleted the total RNA, i.e., the mRNA was split by applying the RNase III enzyme. Subsequently, the splitted mRNA was assorted with adapters following connection. The whole mRNA was converted to cDNA by reverse transcription. Size assortment was performed, and in-gel PCR was executed for library amplification. The extension was confirmed on 2% E-Gel size-select gel. Consequently, the transcriptome library was enumerated by a high-sensitivity DNA chip on a bioanalyzer. The sample was run in triplicate in support of the study of the transcriptome.

### 2.6. Sequencing run, *de novo* assembly and mapping

The execution of the sequencing run was performed by SOLID TOP SEQ-FRAG LIB KIT MM 50 (ThermoFisher Scientific, United States) for the construction of 40–50 million reads for each sample. Subsequent to the quality filtration (where the mean quality score is > = 20) and the adaptor trimming, the higher superiority *A. hydrophila* reads were amassed via the Velvet-Oases pipeline. A total of 9,273 transcript contigs were acquired. A total of 9,273 *A. hydrophila* transcript contigs were recorded on the sorted reads for the removal of misassembled transcript contigs. Following mapping, entire transcript contigs accepted the support conditions and were further employed for functional illustration.

### 2.7. Functional annotation and gene identification

Transcript contigs (9,273) of *A. hydrophila* were then used for functional annotation. The functional annotation was executed by lining up the transcript contigs with the bacterial non-redundant database of NCBI using the BLASTX program. A gene ontology study was performed to identify the significant genes in the *A. hydrophila* transcriptome analysis.

### 2.8. Transcriptome data analysis

A transcriptome data study employed bioinformatics software to obtain complete information on the genes involved in *A. hydrophila.* BLAST (Altschul et al., 1990) was run on the databases of UniProt-TrEMBL as well as Swiss-Prot (Apweiler et al., 2004) in search of the broadly significant genes implicated. GO was initially characterised at different graph intensities on the basis of three important ontologies: biological processes, cellular components, and molecular functions. Gene Ontology interpretations were allocated employing the QuickGO^1^ tool available at the European Bioinformatics Institute (Huntley et al., 2009).

### 2.9. Statistical analysis

Survival data of *Labeo rohita* were arcsine-transformed to satisfy normality and homoscedasticity requirements, as necessary. The data were then subjected to one-way analysis of variance followed by Duncan’s multiple range test using the Statistical Software Statistical Package for the Social Sciences version 24.0. *p*-values smaller at *p* < 0.05.

## 3. Results

### 3.1. Isolation and virulence characterization of bacterial strains

*L. rohita* exhibited hemorrhagic lesion and redness over the body surface which indicates the possible involvement of bacterial pathogen in mortality. Hence, the collected gut samples were processed for bacterial enumeration and characterization. The isolated bacterial strain displayed a Gram-negative character when subjected to Gram staining. Later, the bacterial strain was subjected to 16S rRNA gene and phylogenetic analysis. The amplified 16S rRNA gene from strain were sequenced and submitted to GenBank and the Accession number is OM900178. The BLAST analysis against non-redundant database showed 99.22% identity with *Aeromonas hydrophila* (GenBank Accession Number: MT384379 and CP046954). Afterwards, a phylogenetic tree was prepared with the sequence accessed from NCBI.

We next sought to investigate the virulence of isolated *A. hydrophila* strain possibly involved in the disease development and mortality of *L. rohita*. The control fish did not have mortality or clinical sign of disease during the experimental challenge. However, the fish challenged by intraperitoneal injection with bacterial suspension mostly developed subcutaneous haemorrhagic ulcers of about 0.7–1.8 cm diameter. Reddening at the injection sites and ulcer on the mouth region were also observed.

### 3.2. Total RNA isolation, library preparation and sequencing

**Figure 1:**
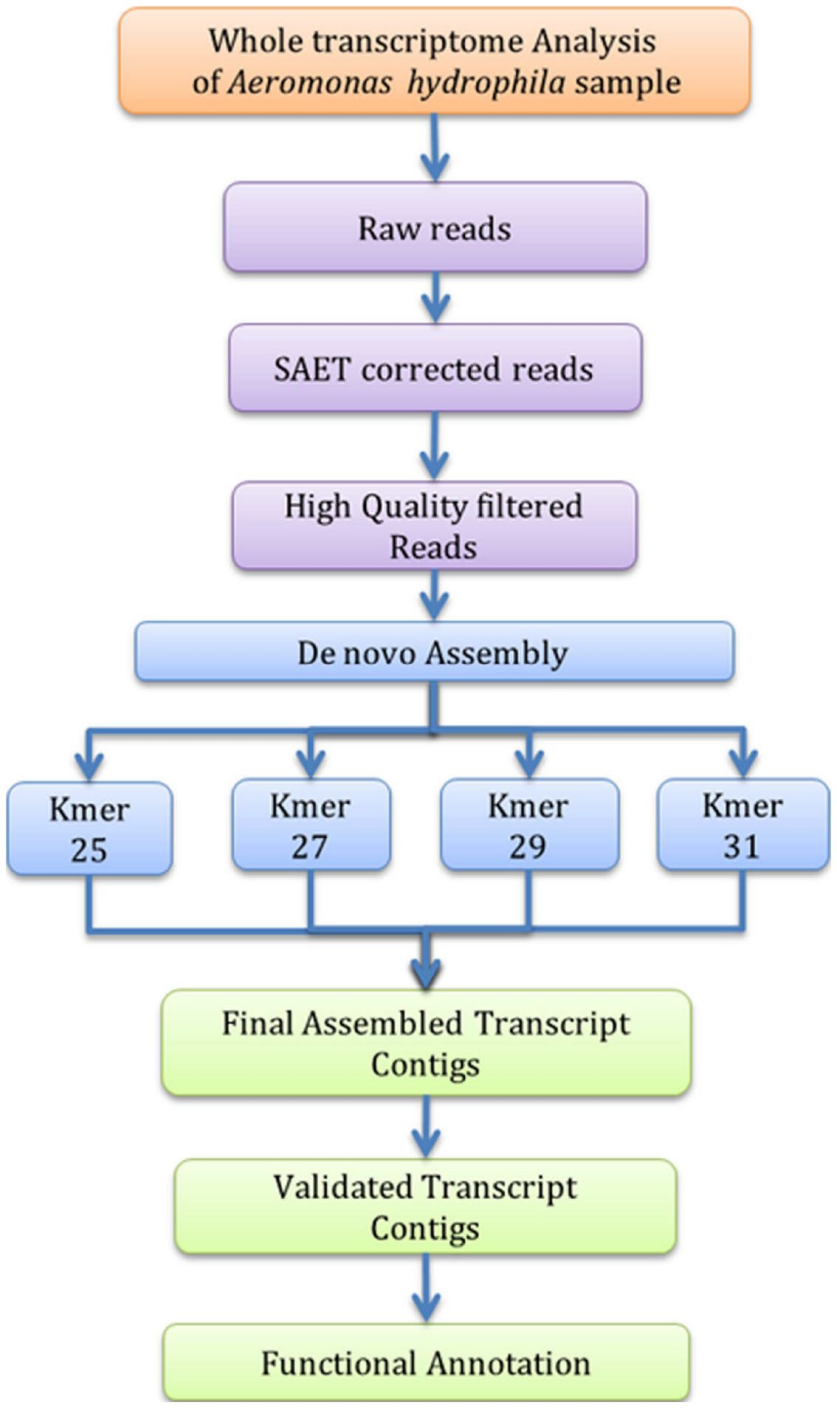
Workflow of *de novo* Transcriptome analysis for *A. hydrophila*.

**Figure 2:**
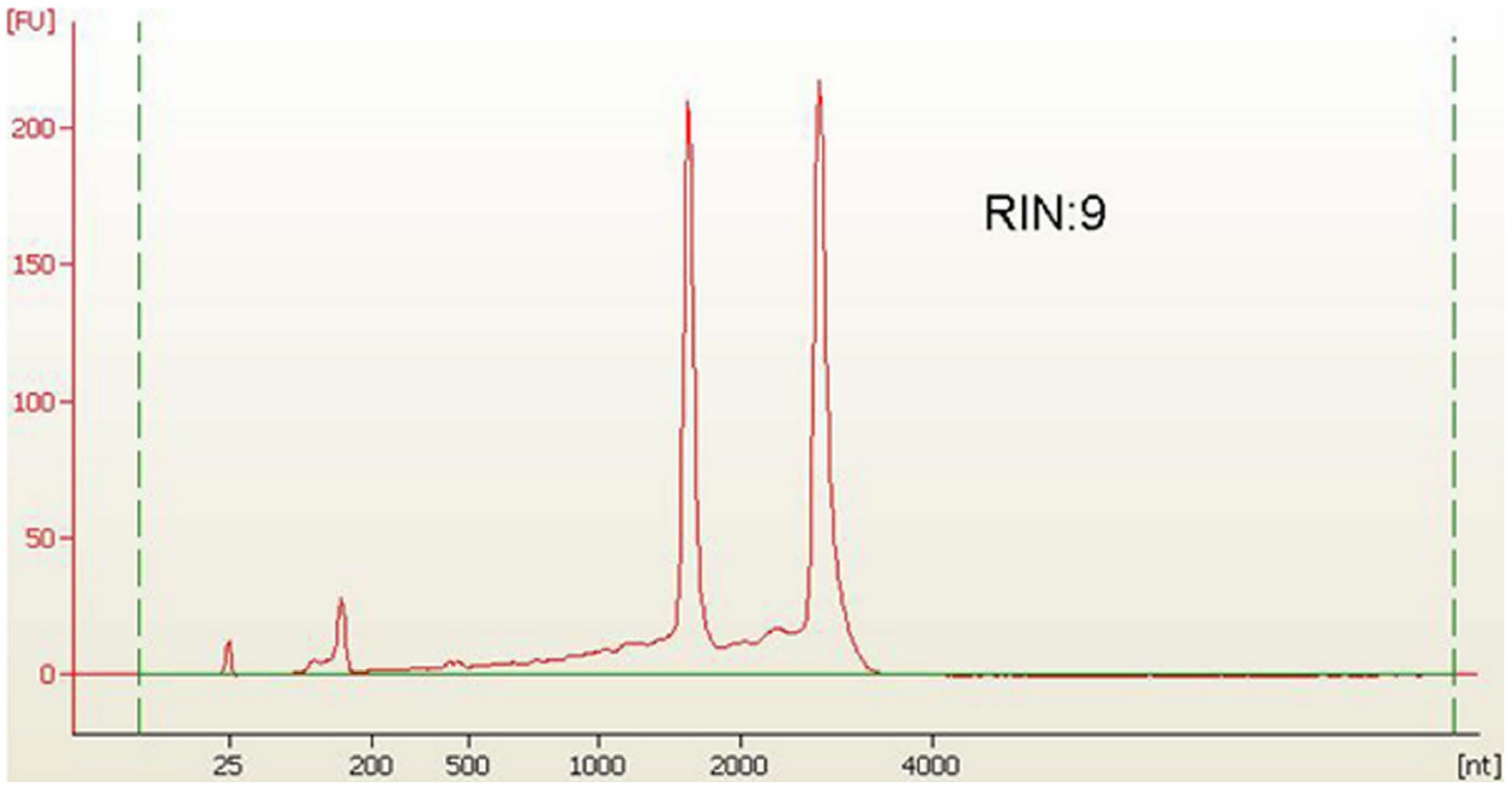
Library profile for *A. hydrophila* on HS Chip.

The *A. hydrophila* isolated from fish gut samples were used for RNA isolation, cDNA and library preparation. The schematic workflow of the whole transcriptome analysis of *A. hydrophila* was depicted (Figure 1). The total RNA integrity was analysed by applying the Bio-Analyzer, which supported the purity of the RNA for further processing (Figure 2). SOLiD sequencing was executed for the transcriptome library preparation of *A. hydrophila.*

### 3.3. Transcriptome assembly and annotation

To prepare the transcriptome of *A. hydrophila*, assembly was executed by applying the entire high-quality reads (after the elimination of replica reads in both samples). Following the quality filtration, i.e., considering the mean quality score > =20 as well as adaptor trimming, the high-quality reads of the *A. hydrophila* sample were accumulated through a *de novo* assembly pipeline employing Velvet v1.2.01 followed by Oases v0.2.04. BLASTX 2.2.26 and Blast2Go 2.5.0 software were used to further analyse the transcriptomic data (Conesa et al., 2005). A sum of 51,461,864 raw reads was produced with 2,573,093,200 numbers of bases with a mean read length of 50 bp, and 41,461,864 filtered reads were generated with 2,073,093,200 numbers of bases with a mean read length of 50 bp. A total of 9,273 transcript contigs were obtained. The assembly statistics and the allocation of transcript contigs conferring their length have been provided in Tables 1, 2. The average GC content of *A. hydrophila* transcript contigs was 52%. *A. hydrophila* has a high fraction of transcript contigs (33.58%) through GC content in an array of 50–55%. The allocation of transcript contigs depending on their length and the GC content distribution has been provided in Supplementary Figures S1, S2. BLASTX hits for every transcript contig reclined inside the cut-off value of 1e-06, and elevated significant resemblances were shown in the transcript contigs. Similarity distribution demonstrated that approximately 80% of transcript contigs had affirmative position lengths involving 50–95% of *A. hydrophila* transcript contigs. The E-value and similarity distribution of *A. hydrophila* have been provided in Supplementary Figures S3, S4.

**Table 1:** Assembly statistics for *A. hydrophila* dataset.

Description	Statistics
Number of transcripts contigs	9,273
Sum of the length of transcript contigs (in base pairs)	2,111,735
Max length of transcript contigs (in base pairs)	4,925
Min length of transcript contigs (in base pairs)	150
Mean length of transcript contigs (in base pairs)	227.73
N50 value (in base pairs)	212

**Table 2:** The distribution of transcript contigs conferring to the length.

Transcript contigs length	Number of the transcript contigs
<50	0
100–200	5,506
200–300	2,575
300–400	648
400–500	235
500–600	122
600–700	59
700–800	34
800–900	20
900–1,000	13
>1,000	61

### 3.4. Functional annotation and annotated data distribution

The *A. hydrophila* 9,273 transcript contigs were recorded on the sorted reads to remove misassembled transcript contigs. A minimum of three plotted reads per transcript contig was required for corroboration of transcript contigs. After mapping, all the transcript contigs approved the confirmation standard and were further used for functional illustration. The *A. hydrophila* 9,273 transcript contigs were subsequently intended for functional annotation. The functional illustration was executed by lining up the transcript contigs with the NCBI non-redundant database of bacteria using the BLASTX program. BLASTX was affected in the illustration of 8,013 transcript contigs out of 9,273. As a result, 1,260 transcript contigs had notable negative BLAST hits. These interpreted transcript contigs were recorded on the GO database, owing to which 6,533 were GO annotated while 1,480 were not allocated any GO terms.

### 3.5. Species distribution study

**Figure 3:**
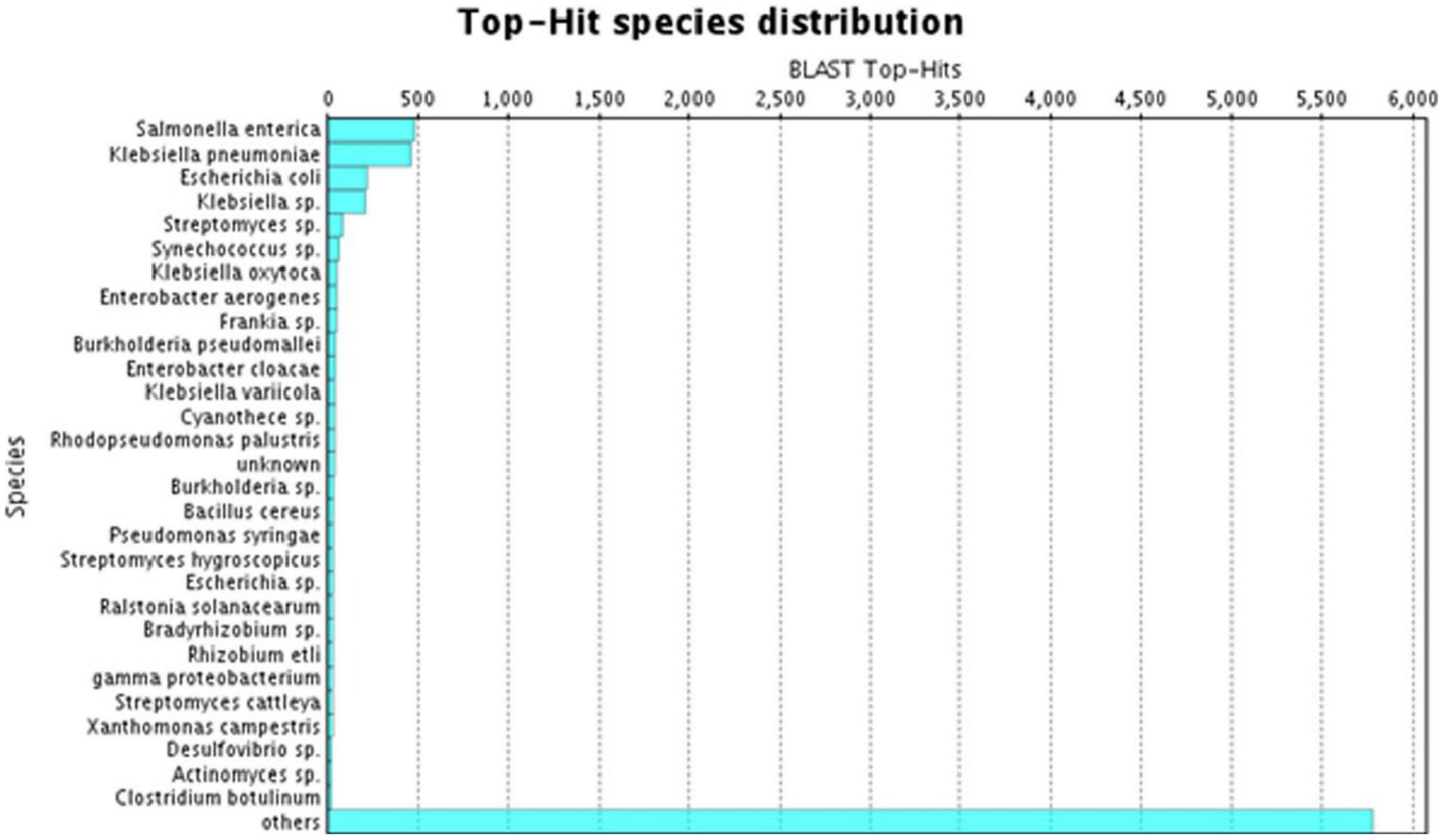
BLAST Top-hit species distribution in *A. hydrophila* transcript contigs.

A species distribution study showed that the utmost proportion of *A. hydrophila* transcript contigs demonstrated momentous resemblance with *Salmonella entrica* followed by *Klebsiella pneumoniae, Escherichia coli, Klebsiella* sp., and *Streptomyces* sp. (Figure 3).

### 3.6. Gene ontology analysis and distribution

**Figure 4:**
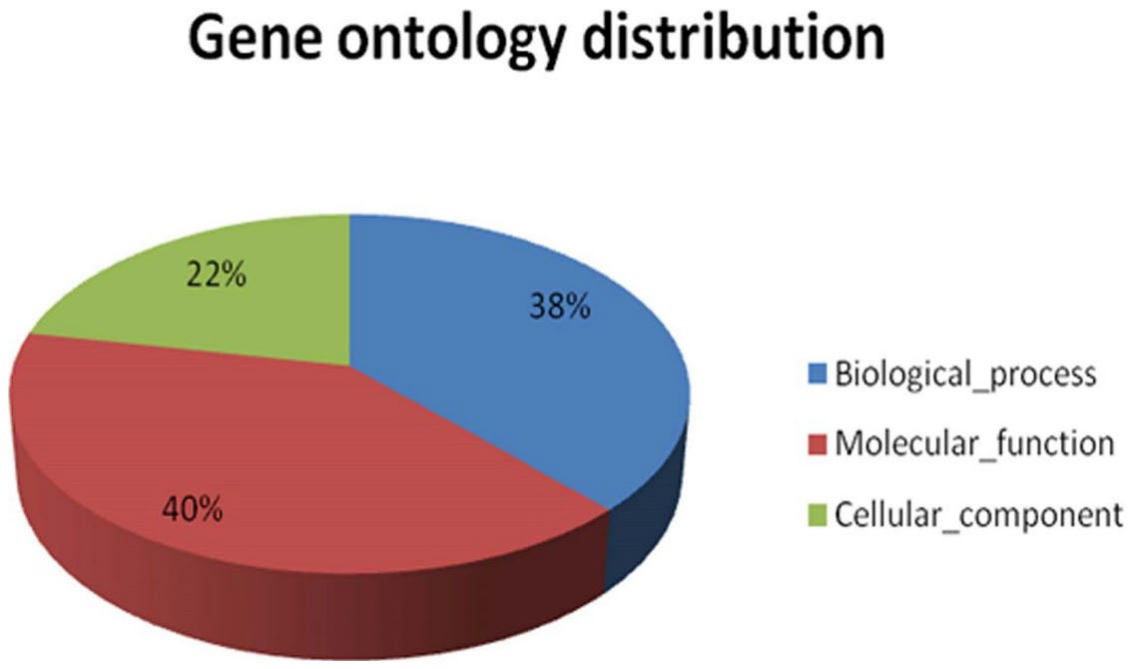
Shows the functional gene ontology distribution for *A. hydrophila* transcript contigs.

GO categorises different functions at different levels of graphs basically on the basis of three main processes, i.e., molecular function, cellular components, and biological processes. The significance of the enrichment is represented by an FDR adjusted value of p. The key illustrates the three areas that Gene Ontology covers, such as molecular activity, biological processes, and cellular components. Only annotations that have a significant false discovery rate (FDR) adjusted value of p of less than 0.05 are shown. In the case of *A. hydrophila*, the cellular component category shows maximum annotations at the 5th level, and the molecular function category shows maximum annotations next to the 7th GO level, while the biological process category shows maximum annotations next to the 6th GO level (Supplementary Figure S5). Gene ontology allocation might be used to better understand the distribution of annotated transcript contigs in precise ontology spheres such as molecular functions, cellular components, or biological processes. Since the total figure of annotated transcripts was 8,013 transcripts, they were mapped to GO. The maximum amount of information in transcript contigs was allocated towards molecular utilities. Individual transcript contigs might be characterised keenly over one GO domain. The transcript contigs allocated for molecular functions are 40, 38% for biological processes, and 22% for cellular components (Figure 4).

### 3.7. Gene ontology sequence distribution

**Figure 5:**
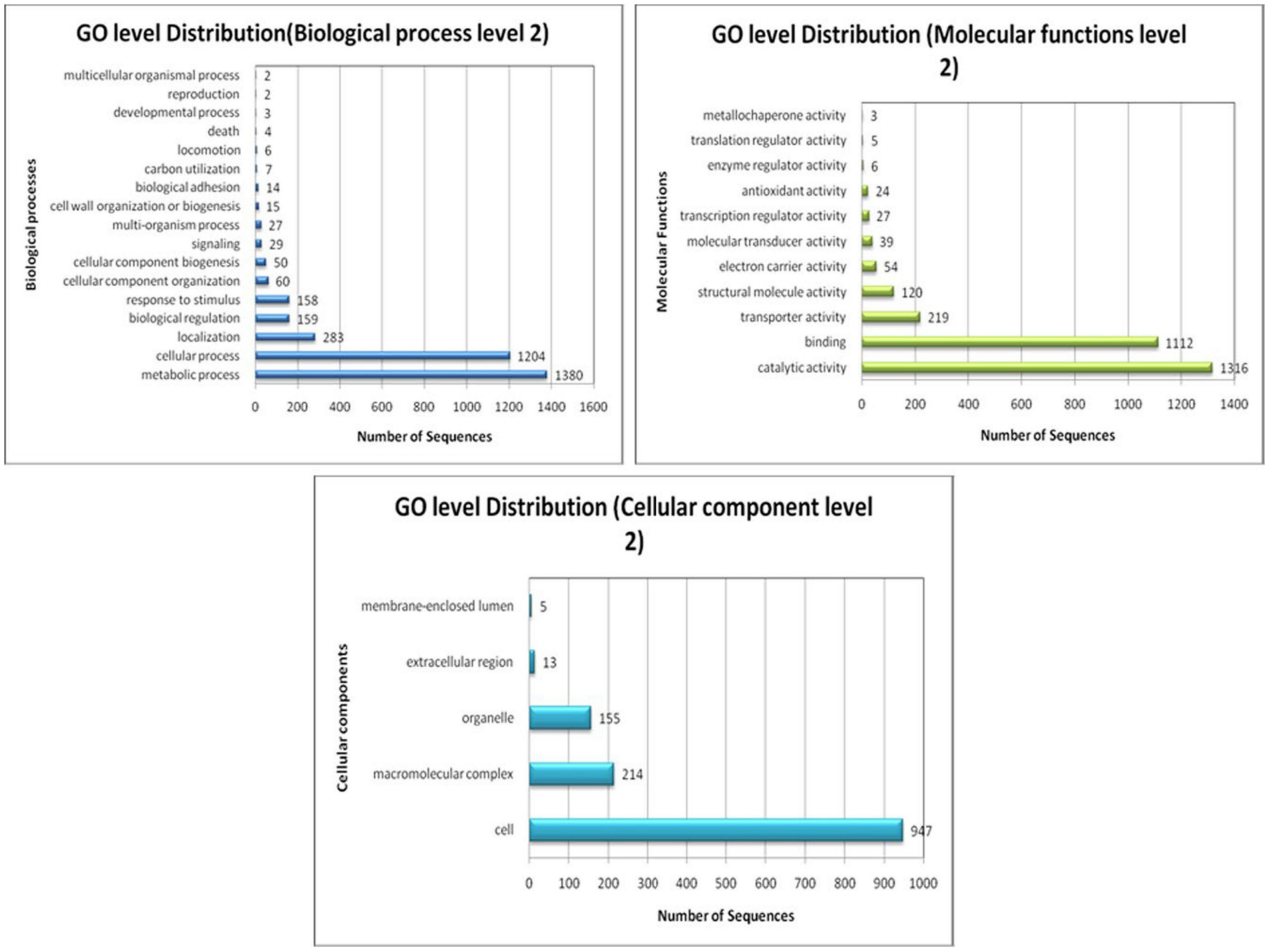
Gene Ontology level 2 sequence distribution of *A. hydrophila*.

GO string allocations help stipulate every interpreted node encompassing GO efficient clusters. Transcripts coupled with comparable tasks are dispensed to identical GO functional clusters. Metabolic and cellular processes are the major biological processes in *A. hydrophila*, since approximately 76% of the transcript contigs in the sample are connected to these ontologies. The main metabolic process includes cellular metabolic processes, oxidation–reduction progression, primary metabolic processes, etc., whereas the main cellular progression includes cellular metabolic processes, cell division, and the cell cycle. Binding activity is the major molecular function within *A. hydrophila* since about 38% of the transcript contigs within the sample are interrelated to this ontology. The binding activity comprises protein binding, nucleotide binding, binding to ions, binding to nucleic acid, binding to cofactors, binding to lipids, binding to vitamins, and so forth. Approximately 50% of transcript contigs are accountable for the catalytic activities. This embraces transferase action, hydrolase action, oxidoreductase action, ligase action, lyase action, isomerase activity, and so forth. The cell is the most important cellular constituent in *A. hydrophila* and composes about 71% of the entire transcript contigs. This comprises the cell part. Around 16% of transcript contigs are related to macromolecular complexes, including protein complexes, protein-DNA complexes, and ribonucleoprotein complexes, while organelles constitute about 12% of transcript contigs. The GO level 2 sequence distribution of *A. hydrophila* is depicted in Figure 5.

### 3.8. Sequence distribution depending on gene ontology study

**Figure 6:**
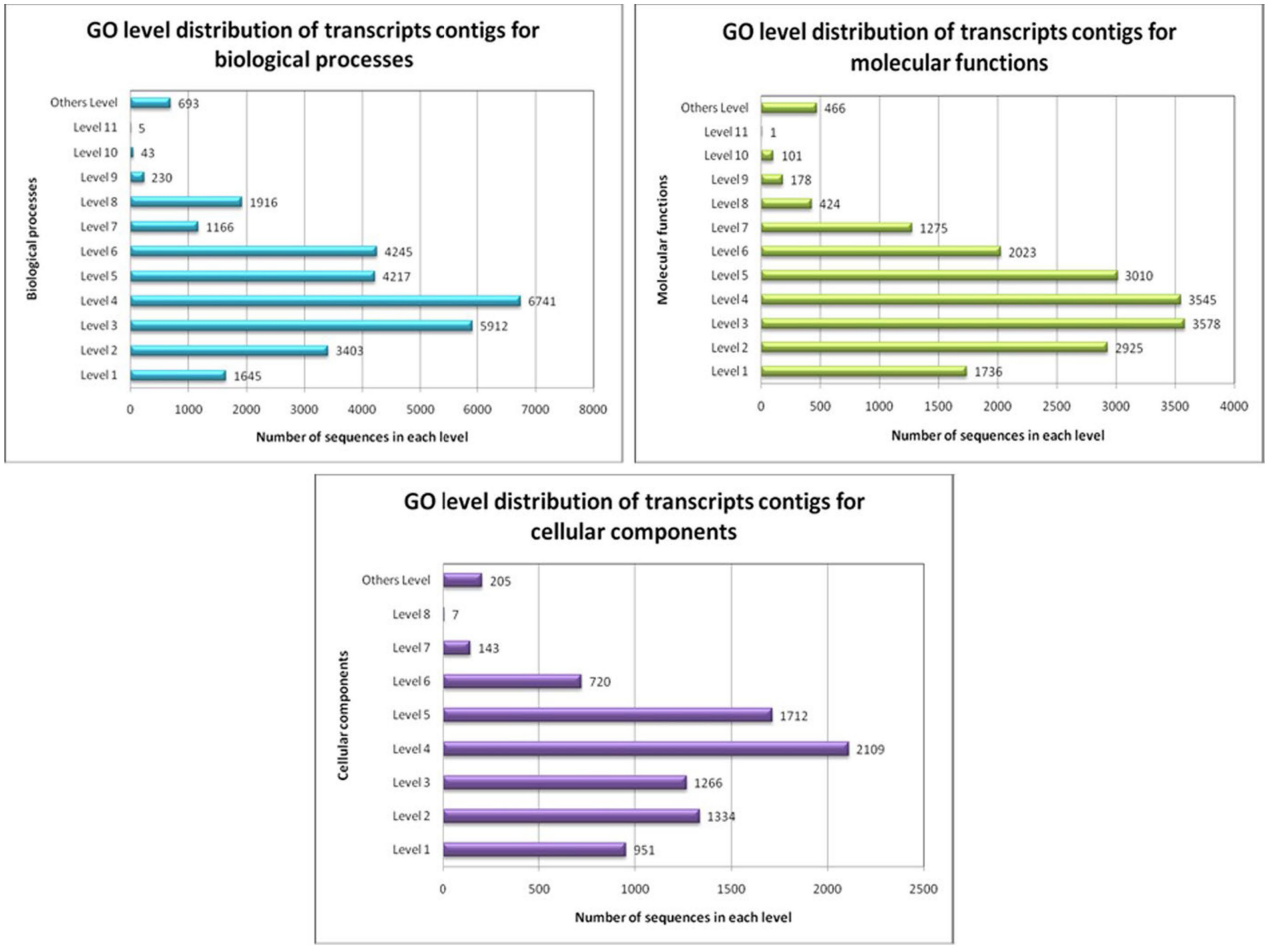
GO-level distribution of transcript contigs in favour of biological processes, molecular functions and cellular components of *A. hydrophila*.

GO conditions were collected into different stages for the three ontology spheres, i.e., the biological processes, the molecular functions, and the cellular components. Preliminary intensities denote the general function of the transcript contigs. When the intensity progresses, the transcript contigs’ role is explicitly added. All transcript contigs might be multi-functional and can recline within in excess of a single function. Therefore, the number of transcripts contigs intended for each stage varied significantly (Figure 6).

### 3.9. Identification of the important genes of *Aeromonas hydrophila*

Several expressed genes have been identified following the transcript annotation and gene ontology analysis of *A. hydrophila*. Different virulent factors of *A. hydrophila* are encoded by several virulent genes. The genes thus identified are the potential virulent genes of *Aeromonas* sp., which contribute to their degree of virulence. The virulent genes include the outer membrane protein gene (ompW, ompA, ompTS, omp 38, omp 48), cytotoxin, amylase lipase, and haemolysin gene (Table 3), which have been reported earlier by Dash et al. (2014). A subset of these virulent genes might be a key player in the ability of the bacterium to cause disease. The products of such genes will facilitate the successful colonisation and survival of the bacterium in or cause damage to the host are considered as virulence or pathogenicity determinants. Hence, these genes can significantly interpret the pathogenic mechanisms exerted by *A. hydrophila* infection.

**Table 3:** Important genes expressed in *A. hydrophila* in definite physiological state.

Gene name	Transcription Id	No. of transcripts
OMP geneA (ompA)	trans_AH_4670, trans_AH_2157, trans_AH_256, trans_AH_536, trans_AH_5928, trans_AH_248, trans_AH_2106, trans_AH_5885, trans_AH_7031, trans_AH_119, trans_AH_1443, trans_AH_116, trans_AH_2401, trans_AH_5139, trans_AH_4745, trans_AH_7450, trans_AH_7532, trans_AH_757, trans_AH_5561, trans_AH_3826, trans_AH_3636, trans_AH_1738, trans_AH_913, trans_AH_427, trans_AH_117, trans_AH_7496	26
OMP gene 38 (omp38) and OMP gene 48 (omp48)	trans_AH_203, trans_AH_7148, trans_AH_256, trans_AH_6727, trans_AH_5928, trans_AH_8576, trans_AH_7619, trans_AH_2058, trans_AH_3513, trans_AH_1297, trans_AH_1537, trans_AH_68, trans_AH_554, trans_AH_2562, trans_AH_831, trans_AH_5574, trans_AH_3277	17
OMP genets (omp TS)	trans_AH_3831, trans_AH_4562, trans_AH_5692	3
OMP geneW (ompW)	trans_AH_1654, trans_AH_1724, trans_AH_1662	3
Amylase gene	trans_AH_7185, trans_AH_6117, trans_AH_3300, trans_AH_7401, trans_AH_6548, trans_AH_3296, trans_AH_8512, trans_AH_8678, trans_AH_8240, trans_AH_6698, trans_AH_4529	11
Lipase gene	trans_AH_8111, trans_AH_4785, trans_AH_4472, trans_AH_6088, trans_AH_6344, trans_AH_1631, trans_AH_7949, trans_AH_2037, trans_AH_5985, trans_AH_7832, trans_AH_4685	11
Haemolysin gene (hlyA)	trans_AH_6097, trans_AH_9044, trans_AH_3696, trans_AH_5237, trans_AH_4015, trans_AH_7216, trans_AH_7846, trans_AH_6957, trans_AH_4754	9
Cytotoxin gene	trans_AH_5207	1

## 4. Discussion

A genome-wide study of bacterial gene expression appears to be progressively variable from microarray tools to second-generation sequencing techniques (Kothari, 2015). Transcriptome profiling is a prevailing advancement to purify the perception of bacterial gene expression. Insufficiencies in complete genome details are hereafter objected to as indications directed to executing transcriptome assembly since the short raw reads use numerous *de novo* assembly programs (Prasath et al., 2014; Razzauti et al., 2015). Amongst the motile Aeromonads, the most frequently bumped into microbial mediator connected to fish infection is *A. hydrophila* (Janda, 1991; O’Brien et al., 1994; Otta et al., 2003), which is accountable for several diseases in cold-blooded animals as well as in warm-blooded animals (Shotts et al., 1976; Shane and Gifford, 1985; Gosling, 1996), leading to substantial economic losses. This study provides for the first time a global view of *A. hydrophila* gene expression profile in fish species during infection. Our data highlights the specific transcriptional pattern produced by *A. hydrophila* and provides new insight in the physiology of this pathogen in the context of fish infections. Moreover, this study allows a better understanding of the temporal activation of metabolic pathways necessary for *A. hydrophila* development. Therefore, taken together, the data effectively raises possibilities to develop new strategies to treat bacterial infections in the context of in farmed fish populations.

Motile Aeromonas septicemia (MAS) caused by *A. hydrophila* causing severe mortality and production losses in aquaculture sector. Our analysis showed that infected fish exhibited clinical signs like extreme lethargy, hemorrhage, ulcer, discoloration, and redness in fins and all over the body surface (Dubey et al., 2022; Semwal et al., 2023). We confirmed both by survival and molecular methods on the involvement of *A. hydrophila* to induce mortality in *L. rohita*. Our transcriptomics analysis investigated the novel *A. hydrophila* transcriptome sequence data and presented through gene ontology. We have also emphasized to identify the significant genes related to immunity. Conversely, the function of the genes concerned with *A. hydrophila* infection as well as mechanisms related to its pathogenicity are mostly unidentified. Consequently, whole genome transcriptomic data were produced to comprehend the *A. hydrophila* infection in an exact ecological condition. The accumulated, interpreted transcriptome sequences and transcript profusion prototypes afford an important genetic reserve for auxiliary explorations of the molecular methods of pathogenicity in this species. Moreover, transcriptome assembly and annotation statistics assist in recognising the entire transcript contigs of *A. hydrophila*. These immunity-related genes include a group of outer membrane protein (OMP) genes, *viz.*, ompA, ompW, ompTS, omp38, omp48, the haemolysin (hlyA) gene, the amylase gene, the lipase gene, and the cytotoxic gene. The information gathered relating to these genes would progress the perceptive study of the pathogenicity of microbial infection.

A considerable amount of microbial transcriptomic data has enriched the database for a couple of years. In addition, studies on the role of some significant genes, both candidate and novel, have been depicted. However, massive research is obligatory to elucidate the precise pathogenic mechanisms essential to microbial infectivity. Among various virulent factors, the outer membrane proteins (OMPs) of Gram-ve microbes play a crucial role in microbial pathogenicity as they are concerned with adaptive microbial reactions similar to multidrug resistance, uptake of iron, antimicrobial peptide resistance, and serum resistance (Lin et al., 2002; Ebanks et al., 2005). However, awfully little information on the *Aeromonas* OMP genes is currently available. In this study, 49 gene transcript contigs related to outer membrane proteins (OMPs) have been obtained, among which 26 gene contigs represent ompA, 17 gene contigs represent omp38 and omp48, three gene contigs belong to ompW, and the rest three belong to ompTS. In addition, 32 other different genes have been obtained, which comprise amylase (11), lipase (11), hemolysin (9), and cytotoxin (1). Seethalakshmi et al. (2008) reported that the disparity in the allocation of prospective antagonistic genes among *A. hydrophila* isolates adds to their degree of antagonism. Therefore, the information obtained from the identified gene contigs can give an insight into the virulent genes related to the pathogenicity of the bacteria. Recent research has accentuated the responsibility of OMPs of virulent bacteria in defensive antigenicity as their uncovered epitopes rest on the cell surface iseffortlessly documented as unfamiliar matters through the immunological protection organisation of the hosts (Kawai et al., 2004). According to Krishnan and Prasadarao (2012), the protein OMPA was primarily recognised in the outer membrane of *Escherichia coli,* but now it has been identified as a primary protein of the outer membrane of an extensive array of Gram-ve bacteria. Primary physiological functions accredited towards the OMPA unit comprise preservation of tectonic integrity, cell morphology, and activity of porin. It also plays a pivotal role in microbial pathogenicity. Consequently, it was once reported as an essential virulent factor in several human and fish pathogens (Smith et al., 2007). Therefore, further analysis of the identified 26 ompA gene contigs thus obtained will give rise to valuable information regarding the functions and pathways related to the ompA gene.

In a related approach, Khushiramani et al. (2007) considered the cloning and expression of the gene encoding for OMP designated as OmpTS of *A. hydrophila* and its immunogenicity within fish. Maiti et al. (2012) evaluated the immunogenicity of OMPW from *A. hydrophila*. Common carp vaccinated amid recombinant ompW and further challenged with *A. hydrophila* illustrated noteworthy antibody assembly and a comparative proportion of endurance of 71%, indicating the defensive effectiveness alongside *A. hydrophila* illness (Maiti et al., 2012). Consequently, in the current study, three gene contigs of ompW and ompTS have been identified, which will be fruitful for getting information related to those genes after further analysis.

In Aeromonads, hly A encodes hemolytic toxin, which plays a significant role in the virulence of the bacterium (Singh et al., 2009). Haemolysin production has been observed as a sturdy indication of virulence in *Aeromonas* sp. (Singh et al., 2008). Sharma and Kumar (2012) detected the hemolysin gene in 15 *A. hydrophila* isolates from the *L. rohita* samples procured from retail markets in Barapani through a multiplex PCR assay. Therefore, in this study, the analysis of the nine hemolysis gene contigs will be invaluable in retrieving information on the functions of hemolysis in bacterial infection.

Kid and Pemberton (2002) cloned and expressed the amylase gene of *A. hydrophila*. Howard and Buckley (1985) reported that lipase is one of the impending virulence genes of *A. hydrophila,* which adds to their measure of pathogenicity. Thus, further analysis of the identified 11 amylase and 11 lipase gene transcript contigs will provide some information regarding the role of these two genes in the proper functioning of the pathways related to the virulence of the bacteria.

Nonetheless, inadequate information is obtainable on the diverse genes concerned with *A. hydrophila* contamination in fish. Within the current research, the transcriptome sequence data of *A. hydrophila* has been analysed, highlighting the finding of biologically essential genes that will escalate our perception of the relationships between genes and the transcriptome of *A. hydrophila*.

It was stated earlier that the outer membrane proteins (OMPs) of the warm water fish pathogen *A. hydrophila* play a significant function in the virulence of these microbes and, in addition, act as a prospective issue for the development of a vaccine to persuade efficient immune defence (Hirst and Ellis, 1994). Therefore, supervising illness nuisance in aquaculture production is a chief alarm ever since aquaculture is one of the fastest-expanding segments of agriculture globally (Das B. K. et al., 2019). In this scenario, discovering new therapeutics is sincerely necessary as microbes are prone to antibiotic resistance. Therefore, the information obtained through the analysis of the gene transcript related to the virulence factors obtained from the *A. hydrophila* whole genome transcriptome will prove to be efficient in discovering some new therapeutic molecules and thus resolving the problem of bacterial diseases in aquaculture to a certain level (Verschuere et al., 2000).

The transcriptome of *A. hydrophila* has been achieved using the SOLiD sequencing platform, collected from the gut of diseased fish *L. rohita*. A couple of significant genes involved in the pathogenicity of microbial infections have been recognised. The transcripts of the identified genes are further examined to enhance the information about the functionalities of the genes. The analysis output affords a profound comprehension keen on the virulent means of *A. hydrophila,* which would be obliging in discovering consistent remedial objects for *A. hydrophila* infection.

## 5. Conclusion

*De novo* whole transcriptome sequencing analysis of *A. hydrophila*, isolated and characterized from moribund diseases *L. rohita*, revealed an activation of a broad range of virulence factors including lipases, toxins and proteases, which are representative of a *A. hydrophila* infection pattern. Interestingly, *A. hydrophila* modified the expression of secretory machineries involved in the release of different virulence factors whose expression were also up-regulated. Taken together these observations support an increased capacity of *A. hydrophila* to provoke overall damage to host tissues. Our observation, therefore, represents an important point in the adaptation of animal research to human medicine. Description of *A. hydrophila* physiology and genome expression during infection in *L. rohita* provides new insights for the understanding of *Aeromonas* infections and appears to be essential to develop new and efficient strategies against pathogenic microorganisms in aquaculture system.

## Data availability statement

The datasets presented in this study can be found in online repositories. The names of the repository/repositories and accession number(s) can be found in the article/Supplementary material.

## Ethics statement

The animal study was approved by Organization for Economic Cooperation and Development (OECD) guidelines. The study was conducted in accordance with the local legislation and institutional requirements.

## Author contributions

BD: conceptualization, funding acquisition, investigation, and supervision. PD: data curation and formal analysis. BD and VK: methodology and writing - original draft. BD and KN: project administration and resources. KN: software. VK and PD: validation. PD and KN: writing - review & editing. All authors contributed to the article and approved the submitted version.

## Conflict of interest

The authors declare that the research was conducted in the absence of any commercial or financial relationships that could be construed as a potential conflict of interest.

## Publisher’s note

All claims expressed in this article are solely those of the authors and do not necessarily represent those of their affiliated organizations, or those of the publisher, the editors and the reviewers. Any product that may be evaluated in this article, or claim that may be made by its manufacturer, is not guaranteed or endorsed by the publisher.

## Supplementary material

The Supplementary material for this article can be found online at: https://www.frontiersin.org/articles/10.3389/fmicb.2023.1247652/full#supplementary-material

## Footnotes

^1^ http://www.ebi.ac.uk/QuickGO/

## Abbreviations

*A. hydrophila*: *Aeromonas hydrophila*
KEGG: Kyoto Encyclopedia of Genes and Genomes
NGS: Next Generation Sequencing
*L. rohita*: *Labeo rohita*
PCR: Polymerase Chain Reaction
SOLiD: Sequencing by Oligonucleotide Ligation and Detection
NCBI: National Center for Biotechnology Information
BLAST: Basic Local Alignment Search Tool
EMBL: European Molecular Biology Laboratory
ORF: Open Reading Frame
RNA: Ribonucleic acid
GC content: Guanine-cytosine content
GO database: Gene ontology database
OMP: Outer membrane protein
