# Supplementary material for: De novo whole transcriptome analysis of Aeromonas hydrophila isolated from the gut of an infected Labeo rohita
Source: Front Microbiol. 2023 Sep 14;14:1247652. doi: 10.3389/fmicb.2023.1247652 (PMC10539578; doi:10.3389/fmicb.2023.1247652)
Supplement: Supplementary file 1 [file Data_Sheet_1.docx]

Figure S1: Distribution of transcript contigs length generated by Velvet and Oases

Figure S2: GC content analysis of *A. hydrophila* transcript contigs.

Figure S3: E-value distribution for *A. hydrophila* transcript contigs

Figure S4: Sequence Similarity distribution for *A. hydrophila* transcript contigs

Figure S5: GO-level distribution for *A. hydrophila* transcript contigs
